# Cigarette Smoke-Induced Collagen Destruction; Key to Chronic Neutrophilic Airway Inflammation?

**DOI:** 10.1371/journal.pone.0055612

**Published:** 2013-01-31

**Authors:** Saskia A. Overbeek, Saskia Braber, Pim J. Koelink, Paul A. J. Henricks, Esmaeil Mortaz, Adele T. LoTam Loi, Patricia L. Jackson, Johan Garssen, Gerry T. M. Wagenaar, Wim Timens, Leo Koenderman, J. Edwin Blalock, Aletta D. Kraneveld, Gert Folkerts

**Affiliations:** 1 Division of Pharmacology, Utrecht Institute for Pharmaceutical Sciences, Faculty of Science, Utrecht University, Utrecht, The Netherlands; 2 Chronic Respiratory Disease Research center, National Research Institute of Tuberculosis and lung disease (NRITLD), Masih Daneshvari Hospital, Shahid Beheshti University of Medical sciences, Tehran, Iran; 3 Department of Respiratory Medicine, University Medical Center Utrecht, Utrecht, The Netherlands; 4 Division of Pulmonary, Allergy and Critical Care Medicine and UAB Lung Health Center, Department of Medicine, University of Alabama at Birmingham, Birmingham, Alabama, United States of America; 5 Danone Research – Centre for Specialised Nutrition, Wageningen, The Netherlands; 6 Department of Pediatrics, Division of Neonatology, Leiden University Medical Center, Leiden, The Netherlands; 7 Department of Pathology and Medical Biology, University Medical Center Groningen, Groningen, The Netherlands; University of Tübingen, Germany

## Abstract

**Background:**

Cigarette smoking induces inflammatory responses in all smokers and is the major risk factor for lung disease such as chronic obstructive pulmonary disease (COPD). In this progressive disease, chronic inflammation in the lung contributes to lung tissue destruction leading to the formation of chemotactic collagen fragments such as N-acetylated Proline-Glycine-Proline (N-ac-PGP). The generation of this tripeptide is mediated by a multistep pathway involving matrix metalloproteases (MMPs) 8 and 9 and prolyl endopeptidase (PE). Here we investigated whether cigarette smoke extract (CSE) stimulates human PMNs to breakdown whole matrix collagen leading to the generation of the chemotactic collagen fragment N-ac-PGP.

**Methodology/Principal Findings:**

Incubating PMNs with CSE led to the release of chemo-attractant CXCL8 and proteases MMP8 and MMP9. PMNs constitutively expressed PE activity as well as PE protein. Incubating CSE-primed PMNs with collagen resulted in collagen breakdown and in N-ac-PGP generation. Incubation of PMNs with the tripeptide N-ac-PGP resulted in the release of CXCL8, MMP8 and MMP9. Moreover, we tested whether PMNs from COPD patients are different from PMNs from healthy donors. Here we show that the intracellular basal PE activity of PMNs from COPD patients increased 25-fold compared to PMNs from healthy donors. Immunohistological staining of human lung tissue for PE showed that besides neutrophils, macrophages and epithelial cells express PE.

**Conclusions:**

This study indicates that neutrophils activated by cigarette smoke extract can breakdown collagen into N-ac-PGP and that this collagen fragment itself can activate neutrophils, which may lead *in vivo* to a self-propagating cycle of neutrophil infiltration, chronic inflammation and lung emphysema. MMP-, PE- or PGP-inhibitors can serve as an attractive therapeutic target and may open new avenues towards effective treatment of COPD.

## Introduction

Chronic inflammation is observed in lung diseases such as chronic obstructive pulmonary disease (COPD) [1]. This disease, referring to bronchitis and emphysema, is an important cause of morbidity worldwide [2], [3] and is characterized by irreversible progressive development of airflow limitation [4]. Neutrophils are a notable component of the inflammation in COPD; they release mediators and proteases, contributing to the chronic inflammatory reaction that ultimately may lead to lung destruction [1], [4]. It is generally accepted that cigarette smoking is the main risk factor for the development of COPD. The World Health Organization estimated that 73% of COPD mortality is related to smoking [5]. Although smoking cessation will beneficially affect disease progression, there is currently no specific therapy for COPD. Since this prevalent disease is an enormous health burden, major efforts have been directed towards understanding the pathophysiology of this complicated disease [2].

One of the most prominent chemokines in COPD is CXCL8. The levels of this chemokine are increased in sputum from COPD patients and correlate with the increased number of neutrophils found in the lungs [1]. Antagonizing CXCL8 with an α-CXCL8 antibody and blocking leukotrienes, such as LTB_4_, with an antagonist incompletely prevents neutrophil chemotaxis in COPD patients [6], suggesting that other chemo-attractants are involved in neutrophil migration in COPD. An example of such a chemo-attractant is N-acetyl-proline-glycine-proline (N-ac-PGP). This tripeptide has been implicated as a new biomarker and therapeutic target for COPD [7]. N-ac-PGP is generated from the breakdown of extracellular matrix collagen and is specifically chemotactic for neutrophils *in vivo* and *in vitro*, as has been shown by different groups [8], [9], [10], [11], [12]. Moreover, chronic airway exposure to N-ac-PGP causes emphysema in mice [12]. In COPD patients, N-ac-PGP was detected in induced sputum samples, whereas this tripeptide was undetectable in healthy individuals and asthmatics [7].

Gaggar *et al.* described the proteolytic cascade that generates the tripeptide PGP from collagen in cystic fibrosis (CF), a disease where chronic neutrophilic inflammation is present in the lungs. Using sputum from CF patients, it was shown that matrix metalloproteases (MMPs) 8 and 9 and prolyl endopeptidase (PE) are involved in this multistep pathway [9].

The aim of this study is to investigate the effect of cigarette smoke on the generation of N-ac-PGP from whole collagen by human neutrophils. Moreover, here we investigated the PE activity in COPD. In this report, we show that neutrophils activated by cigarette smoke extract (CSE) can breakdown collagen into N-ac-PGP and that this collagen fragment itself can activate neutrophils, which may lead to a further increase in neutrophil infiltration, chronic inflammation and lung destruction. Moreover, we propose that PE can play an important role in lung collagen breakdown leading to the development of COPD.

## Results

### Cigarette smoke extract incubation affects cell viability

**Figure 1:**
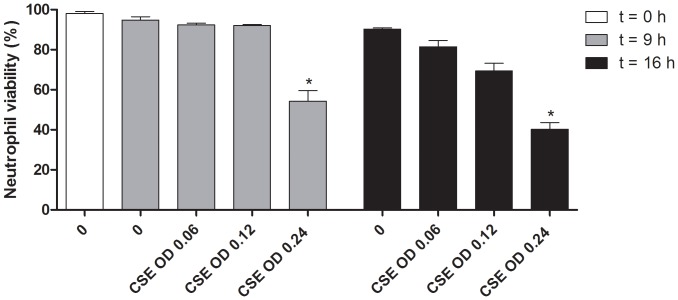
Neutrophil viability after CSE-stimulation for 9 or 16 hours. Significant cell viability loss was observed after 9 and 16 hours incubating neutrophils with CSE OD 0.24 (* *p*<0.05, Friedman, Dunns, n = 3).

Since cigarette smoke consists of more than 4000 compounds known to be mutagenic, carcinogenic, antigenic and cytotoxic [13], [14], the effect on cell viability of cigarette smoke extract (CSE) concentrations used in this study was tested using propidium iodide (PI). Significant cell viability loss was measured after incubating neutrophils with CSE OD 0.24 for 9 hours (*p*<0.05), whereas lower concentrations of CSE were not toxic (Figure 1). This effect was more pronounced after incubating the neutrophils for 16 hours with CSE; CSE OD 0.24 induced a relatively large cell viability loss, whereas lower CSE concentrations showed no significant cell viability loss (Figure 1).

### Incubation of PMNs with CSE induces the release of CXCL8, MMP8 and MMP9

**Figure 2:**
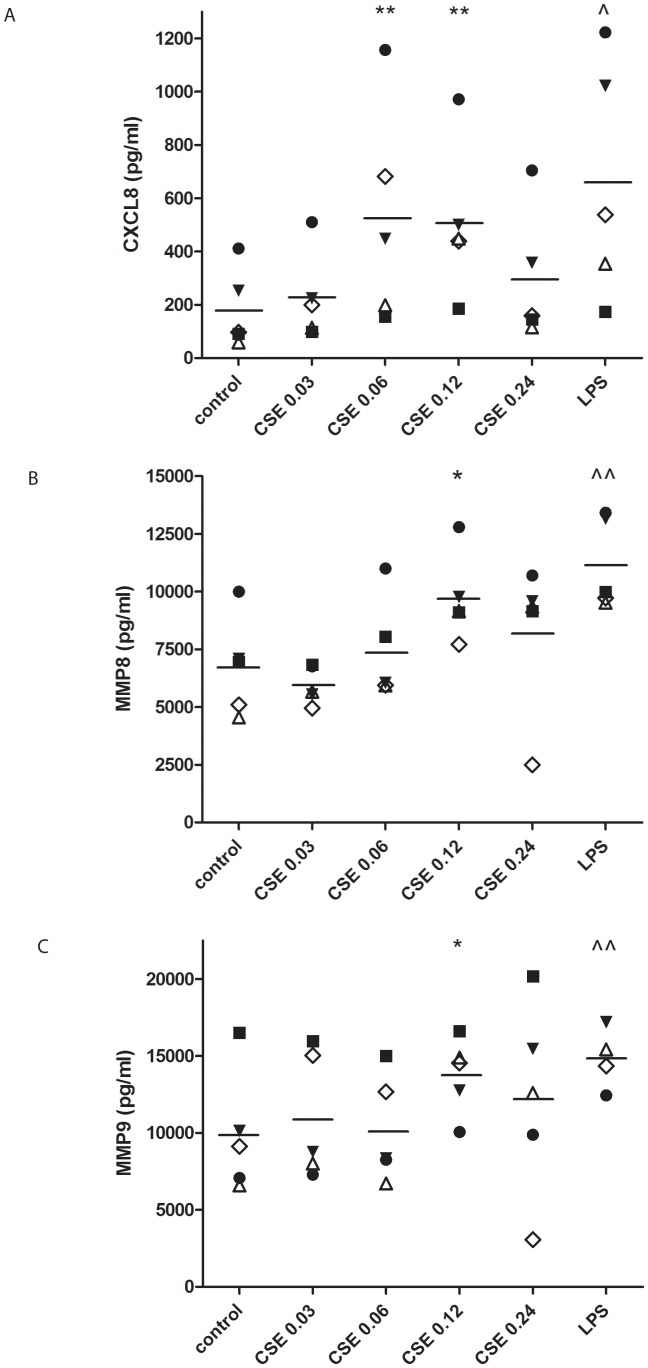
CSE induces the release of CXCL8, MMP8 and MMP9 from human PMNs. (**A**) 10^5^ freshly isolated buffy coat PMNs were stimulated for 9 hours with cigarette smoke extract (CSE; OD 0.03–0.24) or LPS (100 ng/ml; positive control). CXCL8, MMP8 and MMP9 ELISA's were performed on the supernatants. CSE induced the release of CXCL8 from fresh cells (** *p*<0.01 repeated measures ANOVA+Tukey CSE vs. control; ∧ *p*<0.01 paired t-test LPS vs. control). (**B**) CSE induced the release of MMP8 from fresh cells (* *p*<0.05 repeated measures ANOVA+Tukey CSE vs. control; ∧∧ *p*<0.01 t-test LPS vs. control). (**C**) CSE induced the release of MMP9 from fresh cells (* *p*<0.05 paired t-test CSE OD 0.12 vs. control; ∧∧ *p*<0.01 paired t-test LPS vs. control). Legend: each symbol represents a different donor (n = 5). Individual data are shown, horizontal bars represent mean values. The data presented here all passed the normality test.

To examine the role of cigarette smoke in neutrophil activation, PMNs were incubated with CSE for 9 hours. Significantly greater amounts of CXCL8 were produced after stimulation of the cells with CSE OD 0.06 and 0.12 (*p*<0.01; Figure 2A) than in the control, whereas the CXCL8 level produced after 9 hours incubation with CSE OD 0.24 is lower. Incubation of PMNs with increasing concentrations CSE for 9 hours resulted in MMP8 (*p*<0.05; Figure 2B) and MMP9 release (*p*<0 .05; Figure 2C) at CSE OD 0.12. LPS was used as a positive control and induces a significant production of all proteins at 9 hours. In addition, we measured protein release after 16 hours incubation. Similar results were found after 16 hours incubation (data not shown).

### Activity and intracellular levels of PE are unaffected by CSE incubation of PMNs

**Figure 3:**
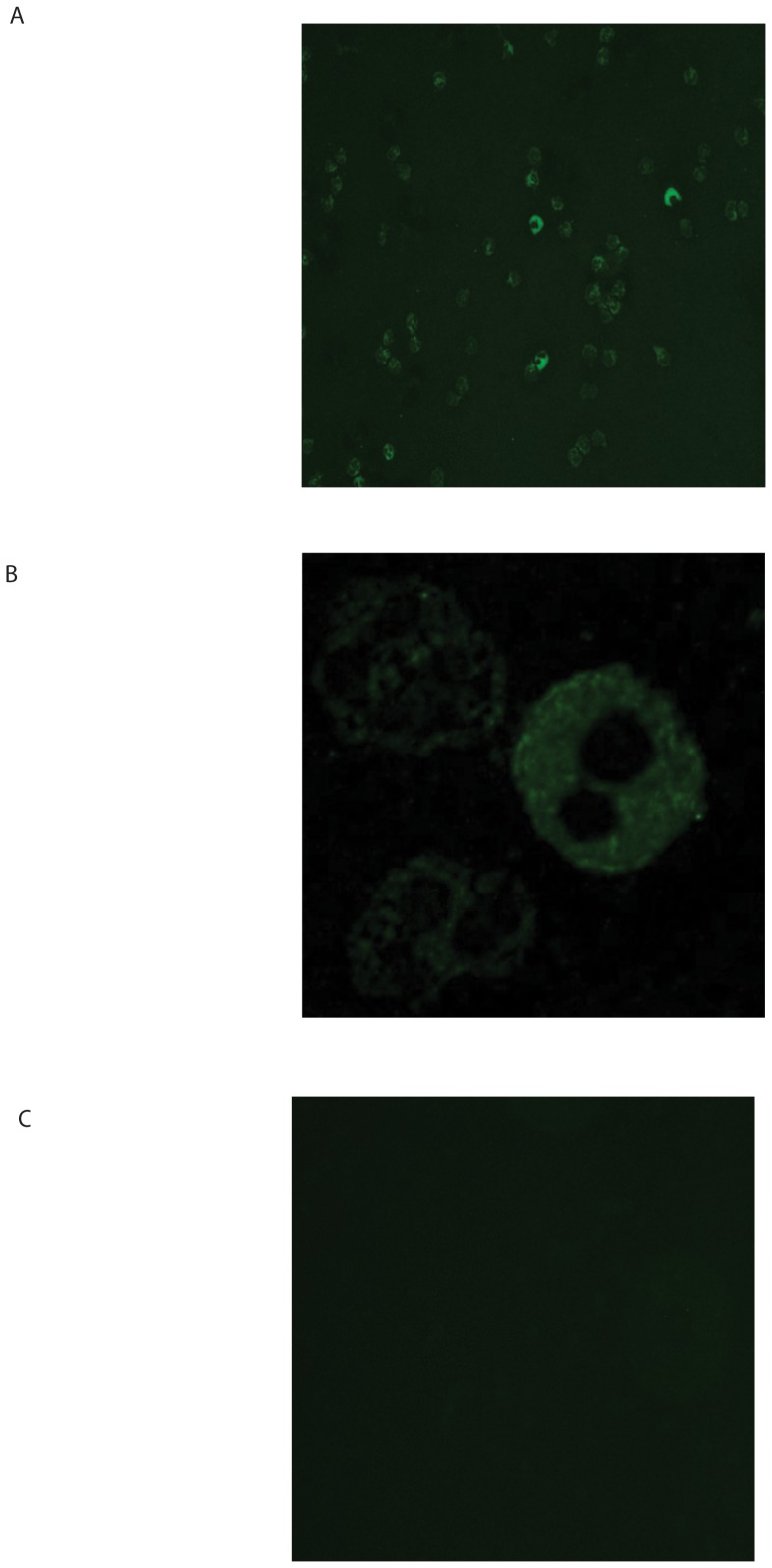
Human PMNs contain prolyl endopeptidase (PE). Representative photomicrographs (n = 3) of an immunofluorescent staining for PE (green color) in PMNs of healthy volunteers (A and B (magnified from figure A)). Panel C displays unstained cells (negative control). PE is located in the cytoplasm in a granular pattern. Magnification 200×.

It has recently been published that neutrophils contain PE [15], an enzyme capable of cleaving the carboxyl side of proline residues in oligopeptides. Detection of PE in PMNs of healthy donors by immunofluorescence microscopy was performed to confirm these results. Figure 3A and 3B show that PE was located in the cytoplasm of PMNs in a granular pattern.

**Figure 4:**
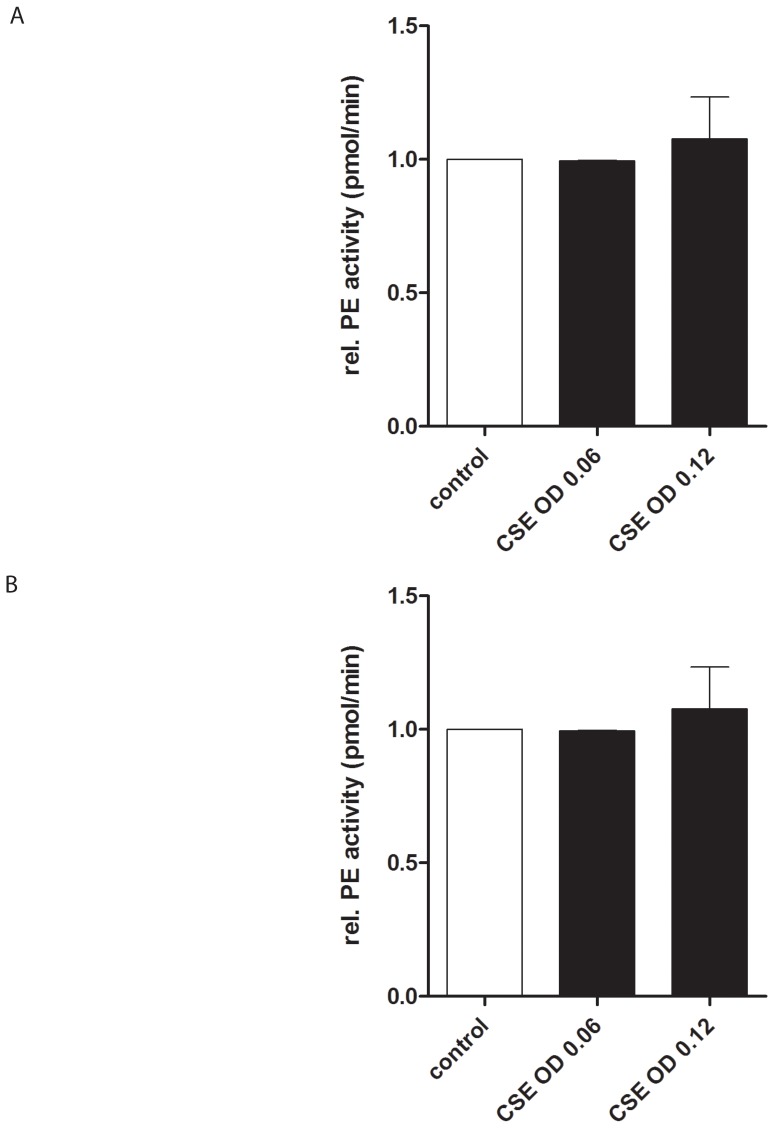
Human PMN incubation with CSE does not affect the level and activity of PE in cell lysates. (**A**) 10^6^ isolated PMNs were stimulated for 9 hours with CSE (OD 0.06 or 0.12). PE and GAPDH Western blots were performed on the cell lysates. PE in human neutrophils was a monomer and migrated at 75 kDa, which was similar to rhPE (not depicted). Incubation of PMNs with CSE did not change the optical density of the bands when compared to the control. (n = 2) (**B**) Freshly isolated PMNs (10^6^ cells) were stimulated for 16 hours with indicated reagents. PE activity was measured in lysates using Z-Gly-Pro-AMC as a substrate. Control was standardized to 1. Intracellular PE activity does not change after CSE exposure when compared to the control. (n = 5).

To investigate whether CSE influences the levels of intracellular PE protein and PE protein in the supernatant, PMNs were incubated with CSE and subsequently PE expression was determined in lysates and supernatants by Western blotting. PE protein levels within the cells did not change after incubation with CSE for 9 hours (figure 4A) and the levels were also unaffected after 1 µg/ml LPS exposure (data not shown). This is not limited to the 9 hour incubation time point; there was no significant difference in PE protein level between the control and CSE-stimulated neutrophils at different incubation times (data not shown). Furthermore, relative intracellular PE activity did not change after CSE incubation for 16 hours (Figure 4B).

Moreover, PE activity was measured in PMN supernatants of healthy donors. PE activity in the supernatant was very low after CSE incubation for 16 hours (control = 0.73 pmol AMC/min; OD 0.06 = 0.27 pmol AMC/min; OD 0.12 = 0.42 pmol AMC/min). Furthermore, PE protein could not be detected in the supernatant of PMNs incubated with CSE or LPS (1 µg/ml) for 9 hours, suggesting that although PMN supernatants contain little PE activity, the protein levels of PE are probably too low to be detected by Western blotting techniques (data not shown).

### Human PMNs can generate N-ac-PGP from whole collagen upon activation with CSE

**Figure 5:**
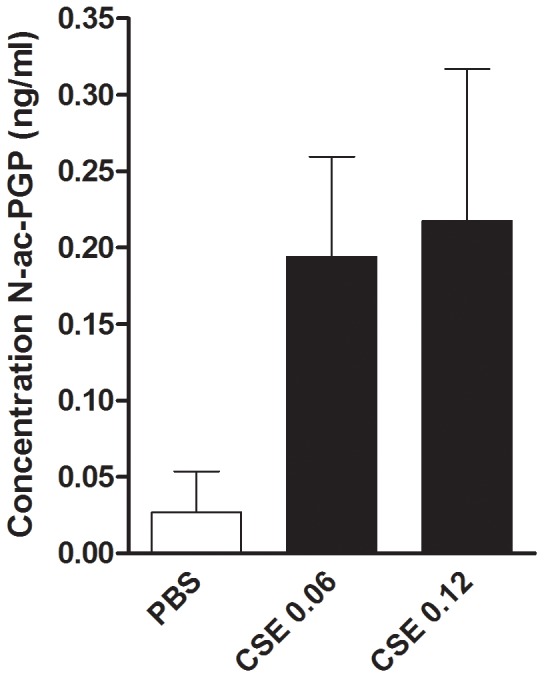
Human PMNs can generate N-ac-PGP de novo from collagen type I. 10^6^ PMNs were incubated for 16 hours at 37°C with collagen type I dialyzed solution (1 mg/ml) and CSE (OD 0.06 or 0.12). Bestatin (50 µg/ml) was added every 3 hours. At time point 16 hours, the N-ac-PGP levels were determined in supernatants from the incubated PMNs. All samples were filtered through a 10-kDa filter, washed with 20 µl of 1 N HCl and analyzed using ESI- LC-MS/MS for levels of N-ac-PGP. Detection limit N-ac-PGP: <0.025 ng/ml. (n = 2).

The data presented in the figures 2, 3 and 4 suggest that cigarette smoke may stimulate neutrophils to breakdown collagen in smaller fragments, and more specifically, to PGP and N-ac-PGP. For these experiments, we used collagen type I since this is the prominent type of collagen seen in the airways [16]. PMNs were incubated with dialyzed collagen type I and CSE (OD 0.06 or 0.12). To prevent any new formed PGP from degrading, bestatin was added every 3 hours. Bestatin inhibits leukotriene A_4_ hydrolase, an enzyme known to degrade PGP [17]. At time point 16 hours, the N-ac-PGP levels were determined in supernatants from PMNs stimulated with PBS or CSE (0.06 and 0.12). CSE OD 0.06 and 0.12 induced a 3–4 fold production of N-ac-PGP from whole collagen type I; the N-ac-PGP levels were 0.194 ng/ml and 0.217 ng/ml respectively (figure 5).

To investigate whether the collagen breakdown process is general to other collagen types, collagen type II was also used. Interestingly, PMNs stimulated with CSE OD 0.06 or 0.12 generated N-ac-PGP levels of 0.217 ng/ml and 0.909 ng/ml, respectively, whereas PBS incubated PMNs did not generate N-ac-PGP levels above detection limit. In addition, the supernatants were examined for non-acetylated PGP levels and were tested negative (data not shown), meaning that all generated PGP is readily acetylated (see discussion).

### N-ac-PGP activates PMNs to release CXCL8 and the proteolytic enzymes MMP8 and MMP9

The ability of PMNs to generate N-ac-PGP from whole collagen upon stimulation with CSE prompted us to investigate whether the peptide itself may activate PMNs to release CXCL8, MMP8 and 9. *In vivo*, this may lead to a self-perpetuating situation where newly produced N-ac-PGP can attract PMNs to the site of inflammation and activate these cells to produce more N-ac-PGP, thereby enhancing inflammation.

**Figure 6:**
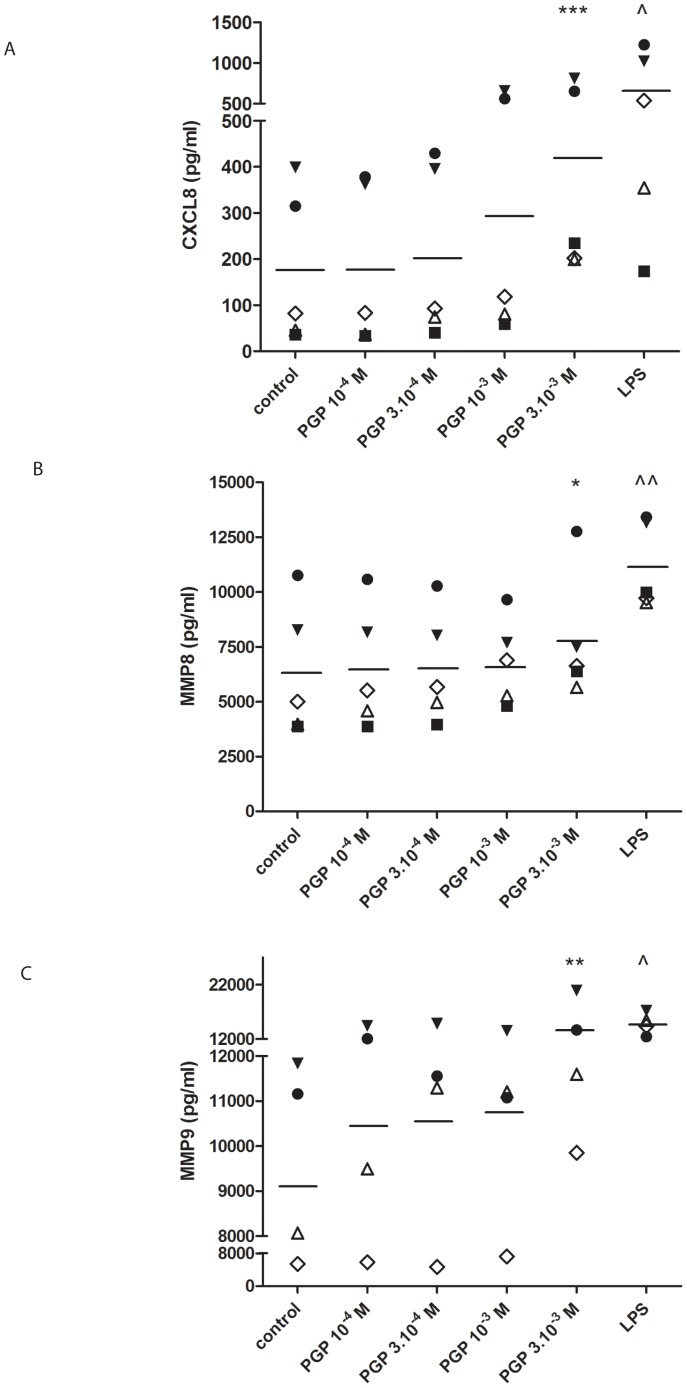
N-ac-PGP induces the release of CXCL8, MMP8 and MMP9 from human PMNs. 10^5^ freshly isolated buffy coat PMNs were stimulated for 9 hours with N-ac-PGP (10^−4^–3·10^−3^ M) or LPS (100 ng/ml). CXCL8, MMP8 and MMP9 ELISA's were performed on the supernatants. (**A**) N-ac-PGP induced the release of CXCL8 from fresh cells (*** *p*<0.001 repeated measures ANOVA+Tukey N-ac-PGP vs. control; ∧ *p*<0.05 paired t-test LPS vs. control). (**B, C**) N-ac-PGP induced the release of MMP8 from fresh cells (* *p*<0.05 paired t-test CSE OD 0.12 vs. control; ∧∧ *p*<0.01 paired t-test LPS vs. control). (**C**) N-ac-PGP induced the release of MMP9 from fresh cells (** *p*<0.01 repeated measures ANOVA+Tukey N-ac-PGP vs. control; ∧ *p*<0.01 paired t-test LPS vs. control). Legend: each symbol represents a different donor (n = 4–5). Individual data are shown, horizontal bars represent mean values. The data presented here all passed the normality test.

PMNs incubated for 9 hours with N-ac-PGP released significantly greater amounts of CXCL8 (*p*<0.001 for 3·10^−3^ M N-ac-PGP; Figure 6A), MMP8 (*p*<0.05 for 3·10^−3^ M N-ac-PGP; Figure 6B) and MMP9 (*p*<0.01 for 3·10^−3^ M N-ac-PGP; Figure 6C) than the control treaded PMNs.

### N-ac-PGP does not affect PE activity in PMNs

**Figure 7:**
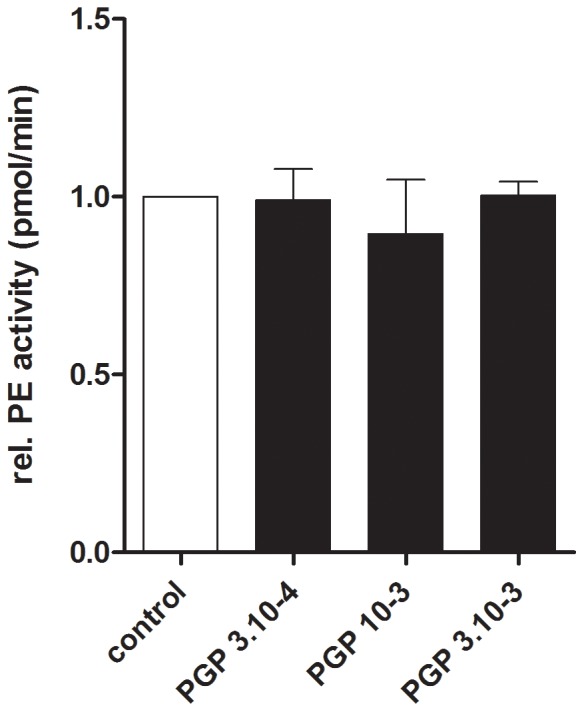
Human PMN incubation with N-ac-PGP does not affect the activity of released or intracellular PE. Freshly isolated PMNs (10^6^ cells) were stimulated for 16 hours with indicated reagents. PE activity was measured in supernatants and lysates using Z-Gly-Pro-AMC as a substrate. Control was standardized to 1. Intracellular PE activity does not change after N-ac-PGP (3·10^−4^–3·10^−3^ M) exposure when compared to the control (n = 3).

To investigate the effect of N-ac-PGP on PE activity, PMNs were incubated with N-ac-PGP for 16 hours and subsequently PE activity was measured. Intracellular PE activity did not change after N-ac-PGP incubation (Figure 7).

Additionally, the PE activity was measured in PMN supernatants of healthy donors. The PE activity measured in the supernatant was very low after N-ac-PGP incubation for 16 hours (control = 0.73 pmol AMC/min; N-ac-PGP 3·10^−4^ M = 0.80 pmol AMC/min; N-ac-PGP 10^−3^ M = 0.58 pmol AMC/min; N-ac-PGP 3·10^−3^ M = 0.44 pmol AMC/min).

### CSE-stimulated PMNs from COPD patients tend to release more CXCL8 than healthy PMNs

**Figure 8:**
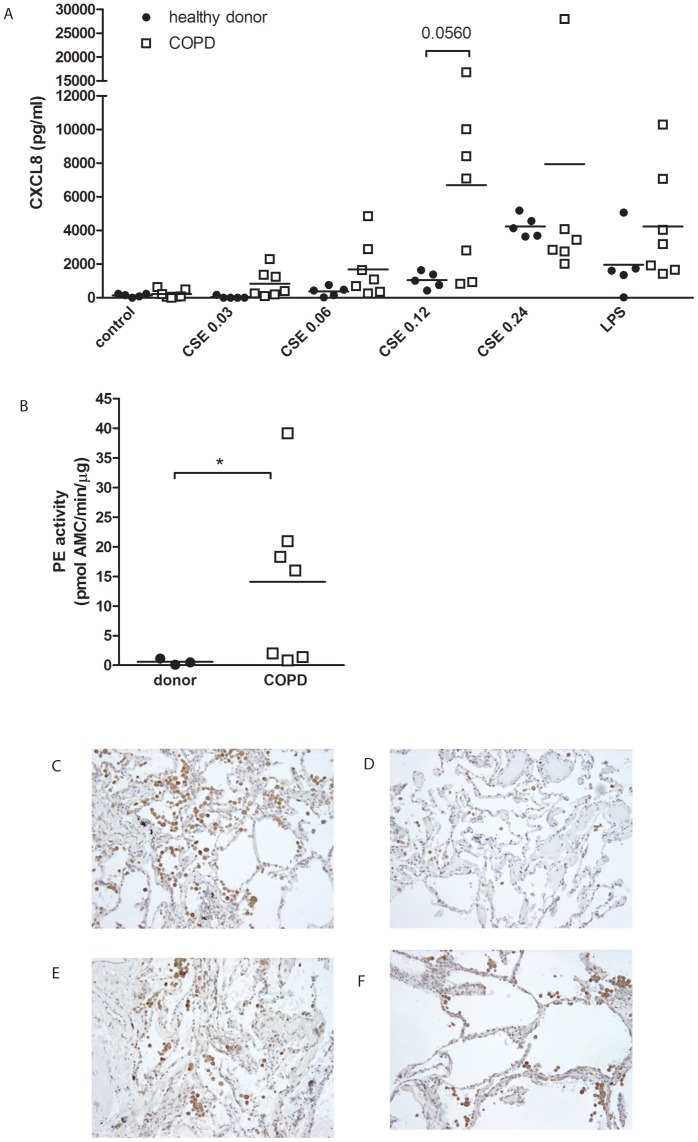
The basal PE activity of PMNs from COPD patients is a 25-fold higher when compared to healthy donors. (**A**) 10^5^ freshly isolated PMNs from healthy donors (block dots, n = 5) and COPD patients (white squares, n = 7) were stimulated for 9 hours with cigarette smoke extract (CSE; OD 0.03–0.24). A CXCL8 ELISA was performed on the supernatants. PMNs from COPD patients tend to produce higher amounts of CXCL8 after CSE incubation (*p* = 0.0560, t-test CSE OD 0.12 donor vs. COPD). Individual data are shown, horizontal bars represent mean values. (**B**) The PE activity was measured in lysates of unstimulated PMNs (10^6^ cells) using Z-Gly-Pro-AMC as a substrate. The basal PE activation of PMNs from COPD patients (white squares, n = 7) is significantly higher than the PE activity of PMNs from healthy donors (block dots, n = 3) (* *p*<0.05 Mann-Whitney). (**C–F**) Localization of PE in the human lung. Representative photomicrographs of an immunohistological staining for PE (brown color, DAB staining) in lung tissue of (**C**) a current smoker, (**D**) ex-smoker, (**E**) COPD patient with GOLD stage II and (F) a COPD patient with GOLD stage IV. Magnification, ×200.

To investigate whether PMNs isolated from fresh blood from COPD patients are intrinsically different from healthy donors, PMNs were exposed for 6 hours to increasing concentrations CSE. Figure 8A shows that PMNs obtained from COPD patients tended to produce more CXCL8 upon stimulation with CSE than PMNs obtained from healthy controls (*p* = 0.056; Figure 8A).

### Basal intracellular PE activity of PMNs from COPD patients is increased compared to healthy donors

The PE activity assay was performed to investigate whether the intracellular PE activity of PMNs from COPD patients is different from healthy donors. Basal PE activation in PMNs obtained from COPD patients was increased 25-fold compared to PMNs from healthy donors (Figure 8B).

### Inflammatory cells in lung tissue of smokers and COPD patients express PE protein

Immunohistochemical analysis was performed to compare the PE expression in lung tissue specimens of current smokers, ex-smokers, COPD patients with GOLD stage II and COPD patients with GOLD stage IV. In the lung tissue specimens of current smokers the massive amount of inflammatory cells, such as neutrophils and macrophages, highly expressed PE (Figure 8C). The number of inflammatory cells and consequently the PE expression was decreased in the lung tissue specimens of ex-smokers (Figure 8D). Furthermore, the inflammatory cells observed in the lung tissue specimens from COPD patients with GOLD stage II and IV also expressed high levels of PE protein (Figures 8E and 8F).

## Discussion

COPD is a lung disease characterized by progressive airflow limitation due to the inflammation-driven destruction of alveolar walls. Neutrophils play a key role in the chronic inflammatory response in COPD. Since cigarette smoke exposure is the main risk factor for the development of COPD we investigated the effect of CSE on the breakdown of whole pulmonary matrix collagen into the chemotactic collagen fragment N-ac-PGP by human neutrophils.

We are the first to show that cigarette smoke can activate neutrophils to generate the chemotactic collagen fragment N-ac-PGP from whole collagen *in vitro*. Incubation of PMNs with CSE activated these cells, which resulted in the release of the chemo-attractant CXCL8 and the proteases MMP8 and MMP9. Simultaneous incubation of PMNs with CSE and collagen resulted in N-ac-PGP generation. In addition, PMNs constitutively expressed PE activity and protein. Simultaneous incubation of PMNs with the tripeptide N-ac-PGP resulted in the release of CXCL8, MMP8 and MMP9.

Moreover, we tested whether PMNs from COPD patients are different from PMNs from healthy donors. Although incubation of PMNs from COPD patients with different CSE concentrations tended to release more CXCL8, this did not reach the level of significance when compared to PMNs of healthy donors. Interestingly, here we show that the intracellular basal PE activity of PMNs from COPD patients is a 25-fold higher when compared to healthy donors. Immunohistological staining of human lung tissue specimens for PE protein showed that besides inflammatory cells, including neutrophils and macrophages also epithelial cells express significant levels of PE protein.

Early in inflammation, neutrophils migrate from the capillaries into the interstitial space, following a chemotactic gradient of CXCL8 [18]. At the site of inflammation neutrophils are activated, leading to the release of more CXCL8 [1], [19]. This release leads to a self-perpetuating inflammatory state where neutrophils attract more neutrophils via chemokine receptors CXCR1 and CXCR2 [20], [21], [22]. Recently, we showed that cigarette smoke extract (CSE) can act as a chemo-attractant for PMNs [23]. This led to the question whether CSE may activate the neutrophil to synthesize CXCL8, acting in an autocrine/paracrine fashion. Figure 2 shows that the activation of PMNs by CSE exposure leads to the release CXCL8. We hypothesize that once infiltrated in the lung tissue, cigarette smoke activates the infiltrated neutrophils. This activation results in a CXCL8 release by the neutrophils, which in turn will attract more neutrophils into the airways.

The increased expression of MMPs is considered to be a key factor in the development of COPD. In this study, the MMP8 and MMP9 release by PMNs was elevated after cigarette smoke and N-ac-PGP exposure to human neutrophils. These results are in accordance with clinical data from different groups. It was shown that although MMP8 and MMP9 levels are lower in smokers when compared to COPD patients [24], [25], the MMP levels from both groups are elevated when compared to non-smokers [24], [25], [26], [27]. Here we show that CSE-stimulated COPD neutrophils did not produce more MMP-9 in comparison to the neutrophils of healthy donors (Figure S1). However, it has been published that COPD patients have higher neutrophil counts in the bronchoalveolar lavage fluid [24], [27], [28]. This leads to the conclusion that the increase in MMP9 levels in COPD patients is the result of an increase in neutrophil number and not due to an increase in MMP9 release.Besides MMP8 and MMP9, PE is needed to generate PGP from whole collagen; the MMPs cleave whole collagen in fragments of 30 to 100 amino acids in length, after which PE specifically cleaves PGP from these smaller fragments [9]. Recently, it was published that neutrophils contain PE [15], which is confirmed in this study. PE activity was measured in lysates of PMNs. Incubation of PMNs with CSE or N-ac-PGP did not affect intracellular PE activity, which suggests that PE is constitutively active. Although PE activity could be measured in the supernatant of CSE or N-ac-PGP incubated PMNs, these levels were very low. We hypothesize that cigarette smoking causes a locally restricted lung inflammation where necrotic neutrophils or neutrophils undergoing NETosis release PE to the exterior, which contributes to PGP generation. This can be substantiated with data from figures 1 and 5; incubating PMNs for 16 hours with CSE resulted in a decrease in cell viability, PE release and subsequent generation of N-ac-PGP from whole collagen. It is possible that other cells besides neutrophils play a role in collagen destruction by supplying PE. Figure 7 shows that also pulmonary alveolar macrophages express PE. Neutrophils and macrophages present in lung tissue of current smokers and COPD patients with GOLD stage II and IV highly expressed PE, while the number of inflammatory cells and consequently the PE expression was decreased in the lung tissue of ex-smokers.

The next step was to investigate the effect of CSE on the breakdown of whole collagen into collagen fragment N-ac-PGP by human neutrophils. The multistep pathway of collagen breakdown has been studied in a murine model of cigarette smoke-induced lung emphysema in our group by Braber et al. [29]. There it was demonstrated that all relevant components (neutrophils, MMP8, MMP9 and PE) involved in this pathway to generate (N-ac-)PGP from collagen were upregulated in the airways exposed to cigarette smoke, suggesting that activation of cells by cigarette smoke leads to the release of proteases and extracellular matrix breakdown. Although this murine model showed that (N-ac-)PGP is formed after cigarette smoke exposure in the airways, here we demonstrate using *in vitro* techniques that upon stimulation with CSE the human neutrophil is able to breakdown collagen into N-ac-PGP fragments. Our results confirm the findings of O'Reilly *et al*, who found that *in vitro* human neutrophils were capable to generate PGP from whole collagen after LPS exposure [15].

Neutrophils contain all necessary components for PGP generation and in this report we demonstrated that simultaneous incubation of these cells with CSE and collagen leads to PGP generation. Although N-ac-PGP levels were measurable after a 16 hour incubation period, non-acetylated PGP could not be detected in these supernatants. This can be explained by the fact that cigarette smoke itself is responsible for N-terminally acetylating PGP, thereby enhancing its chemotactic capacity [17].

Louhelainen et al. [26] and Miller et al. [30] showed that smoking cessation improved lung function although elevated neutrophil counts and the protease burden in the airways continued for months. An explanation for the elevated neutrophil influx and protease levels after smoke cessation is that the continued neutrophil chemotaxis and activation is mediated via N-ac-PGP. In this study we demonstrate that this chemotactic tripeptide can activate neutrophils to release CXCL8 that will lead to an increase in neutrophilic migration. In addition, N-ac-PGP also induced the release of MMP8 and MMP9 from neutrophils, which will result in more collagen breakdown and formation of N-ac-PGP. It was recently published that N-ac-PGP can induce the release of MMP9, which is confirmed in this study. It was indicated that extracellular matrix-derived N-ac-PGP could result in a feed-forward cycle by releasing MMP-9 from activated PMNs through the ligation of CXCR1 and CXCR2 and subsequent activation of the ERK1/2 MAPK [31].

Tissue destruction is a hallmark of COPD. Since PE is essential in the collagen breakdown process, we measured the basal intracellular PE activity in PMNs from COPD patients. Interestingly, the basal PE activity of PMNs from COPD patients was remarkably higher than in PMNs from healthy donors, which suggests that PE can play an important role in lung collagen breakdown leading to the development of COPD. This phenomenon is not limited to COPD; Gaggar *et al.* also observed that the PE activity was elevated in cystic fibrosis samples when compared with normal controls [9]. Moreover, here we suggest that the PMNs from COPD patients are activated to a greater extend, since the CXCL8 levels released by these PMNs appeared to be higher than from PMNs from healthy controls.

Finally, to our knowledge, this is the first *in vitro* study that indicates that neutrophils activated by cigarette smoke can destruct collagen into N-ac-PGP and that this collagen fragment can activate neutrophils, which may lead *in vivo* to a self-propagating cycle of neutrophil infiltration, chronic inflammation and lung emphysema.

From this study but also from other studies on collagen breakdown in COPD mice models [8], [29], we can conclude that different treatment interventions are possible to tackle the ongoing inflammation observed in COPD. MMP inhibitors might be a valuable drug target; in addition to suppressing the accelerated extracellular matrix turnover, the generation of chemotactic N-ac-PGP can be counteracted. Since we show in figure 8 that the basal intracellular PE activity of neutrophils is a 25-fold higher in COPD neutrophils in comparison to the neutrophils from healthy donor PE inhibitors such as valproic acid (VPA) might be useful. This has already been tested in an animal model; a significant decreased neutrophil influx in the BAL fluid of smoke-exposed mice was observed after treatment with VPA [32]. Also, treatment with peptide L-arginine-threonine-arginine (RTR), which binds to PGP sequences, led to a decrease in neutrophil migration in mice exposed to smoke [29].

In conclusion, MMP-, PE- or PGP-inhibitors can serve as an attractive therapeutic target and may open new avenues towards effective treatment of COPD.

## Materials and Methods

### Ethics Statement

Human polymorphonuclear leukocytes (PMNs) were obtained from healthy adult volunteers and COPD GOLD I–III patients. This protocol was approved by the University Medical Center Utrecht Review Board for Biomedical Research. In addition, written informed consent was provided by each study participant.

Tissue specimens were obtained from patients undergoing resective surgery for pulmonary carcinoma or lung transplantation for severe COPD (see further). The study protocol was consistent with the Research Code of the University Medical Center Groningen (http://www.rug.nl/umcg/onderzoek/researchcode/index) and national ethical and professional guidelines (“Code of proper secondary use of human tissue”; Dutch federation of biomedical scientific societies”; htttp://www.federa.org). The data was coded to de-identify the samples accordingly.

### Chemicals and reagents

2R4F reference cigarettes were from Kentucky Tobacco Research Institute (Lexington, KY, USA). Recombinant human CXCL8 and PE, human MMP8 and MMP9 ELISA kits and rabbit IgG antibodies were supplied by R&D Systems Europe Ltd. (Abingdon, United Kingdom). Z-Gly-Pro-7-amido-4-methylcoumarin (Z-G-P-AMC) was purchased from Bachem. LPS, BSA, Triton ×100, diaminobenzidene, selenite, Hoechst stain solution and collagen type I and II were purchased from Sigma Aldrich Chemie BV (Zwijndrecht, the Netherlands). The human CXCL8 ELISA kit was from BD Biosciences (Alphen a/d Rijn, the Netherlands). HEPES was obtained from Agros Organics (Geel, Belgium). Mayers' haematoxylin, H_2_O_2_, NaCl, KCl, K_2_HPO_4_·3H_2_O, CaCl_2_, NH_4_Cl, KHCO_3_, EDTA (Triplex III) and trisodium citrate dihydrate were purchased from Merck KGaA (Darmstadt, Germany). Ficoll-Paque™ PLUS was purchased from GE Healthcare (Eindhoven, the Netherlands). FITC-labeled goat anti-rabbit secondary antibody was purchased from Southern Biotechnology (Birmingham, AL, USA), whereas the rabbit anti-human PE antibody was from ProteinTech Group (Manchester, UK), the 5% goat serum and the biotinylated secondary antibody goat-anti-rabbit from Dakocytomation (Glostrup, Denmark) and the goat anti-rabbit-HRP antibody from DAKO (Enschede, the Netherlands). Vectastain Elite ABC was obtained from Vector Laboratories (Burlingame, USA). Permount was from Fisher Scientific. PBS and Roswell Park Memorial Institute (RPMI) 1640 medium (without L-glutamine and phenol red) were obtained from Lonza Verviers SPRL (Verviers, Belgium). FBS was from Perbio Science Nederland BV (Etten-Leur, the Netherlands).

### Lung tissue specimens

The characteristics of the human subjects included in the study are presented in Table 1.

**Table 1:** Characteristics of COPD patients and controls (lung tissue).

	CURRENT SMOKER	EX-SMOKER	COPD PATIENT GOLD STAGE II	COPD PATIENT GOLD STAGE IV
**Gender (m/f, n)**	0/6	0/6	5/3	0/6
**Age (yrs)**	58 (49–67)	59 (51–67)	67 (58–76)	54 (49–60)
**Current smoker/not current smoker (n/n)**	6/0	0/6	4/4	0/6
**Packyears (yrs)**	30 (25–45)	31 (18–44)	38 (21–55)	34 (21–47)
**FEV_1_, % predicted**	94.2 (84.7–103.7)	88.2 (83.2–93.2)	65.6 (55.6–75.6)	19.5 (13.7–25.3)
**FEV_1_/FVC, %**	78.4 (71.0–85.8)	71.9 (62.9–80.9)	58.7 (50.2–67.2)	35.6 (20.9–50.3)

*Data are presented as median (range). FEV1: Forced Expiratory Volume in one second. FVC: Forced Vital Capacity. The FEV1 of the current smoker and ex-smoker is based on prebronchodilator values and the FEV1 of the COPD patients is based on post-bronchodilator values.*

Tissue specimens from the current smokers and ex-smokers were obtained from noninvolved lung tissue from patients undergoing resective surgery for pulmonary carcinoma. These patients had no airway obstruction and no chronic airway symptoms, such as cough and sputum production. Material was always taken from as far away as possible from the tumor, or from a noninvolved lobe. No histopathological lesions were present in these tissue specimens.

Tissue specimens of GOLD stage II COPD patients were obtained from noninvolved lung tissue from patients undergoing resective surgery for pulmonary carcinoma. Tissue was always taken as far away as possible from the tumor, or from a noninvolved lobe. Histopathologically emphysematous lesions were present in these tissue specimens, however, of limited but varying severity. The moderate forms can be histopathologically demonstrated by the finding of isolated or freelying segments of viable alveolar septal tissue or isolated cross sections of pulmonary vessels.

Tissue specimens of GOLD stage IV COPD patients were obtained from patients with COPD undergoing surgery for lung transplantation or lung volume reduction. All these patients had to quit smoking for at least 1 yr before surgery. The resected tissue showed both macroscopically and microscopically severe emphysematous lesions, often accompanied by bullae. Subpleural, fibrous areas were avoided. Immunohistochemistry was performed on 3 µm formalin-fixed, paraffin-embedded lung tissue.

### Human polymorphonuclear leukocytes

Human polymorphonuclear leukocytes (PMNs) were isolated as previously described from fresh whole blood [10], for which healthy donors signed written informed consent forms or from buffy coats, which were purchased from Sanquin Blood Bank (Amsterdam, The Netherlands). Resulting PMN preparations consisted of 95–97% PMNs, based on PMNs physical parameters analyzed by flow cytometry and CD16 expression. The preparations were negative for CD14, meaning that the preparations did not contain monocytes.

PMNs from COPD patients were collected and were tested for CXCL8 release and PE activity. For this PMN study, the PMNs from fresh whole blood of healthy donors and COPD patients were used. The characteristics of the human subjects included in the PMN study are presented in Table 2.

**Table 2:** Characteristics of COPD patients and healthy controls (PMNs).

	HEALTHY DONOR	COPD PATIENT GOLD STAGE I–III
**Gender (m/f, n)**	3/5	7/4
**Age (yrs)**	45 (26–63)	60 (35–72)
**Current smoker/not current smoker (n/n)**	0/8	5/6
**Packyears (yrs)**	-	31 (22–67)
**FEV_1_, % predicted**	-	55.7 (27.9–99.9)
**FEV_1_/FVC, %**	-	47 (27–70)

*Data are presented as median (range).*

### Cigarette smoke extract (CSE)

CSE was prepared by using a smoking machine (Teague Enterprises, Davis, Ca, USA) as previously described [33]. Direct and side stream smoke from one 2R4F cigarette was directed via a tube through 5 ml PBS using a peristaltic pump. The optical density (OD) of this extract was determined using a spectrometer (UV-mini 1240, Shimadzu) measuring at wavelength 320 nm. Freshly prepared CSE was used in all experiments. Non-toxic solutions ranging from 0.03 to 0.24 OD were used in the present study as determined by Annexin-V staining and FACS analysis.

### Assessing cell viability

Cell viability was assessed on fresh cells at the end of each treatment with propidium idodide (PI, Sigma-Alderich) staining. 10,000 events were counted in the cell gate by FACS (FACS Calibur, BD Bioscience, Becton Dickinson).

### CXCL8, MMP8 and MMP9 ELISA's

PMNs (10^5^ cells/well) were incubated for several time points with indicated reagents. After incubation, the supernatants were collected and CXCL8, MMP8 and MMP9 levels were measured using a human CXCL8, MMP8 or MMP9 ELISA kit according to manufacturer's instructions.

### Immunofluorescence microscopy

Cytospin preparations of PMNs on glass slides were fixed with 4% paraformaldehyde in PBS and permeabilized with 0.1% Triton X-100. After blocking with 3% BSA in PBS, PMNs were incubated with anti-PE antibody (45 µg/ml in PBS/1% BSA), pre-immune rabbit antibody or anti-PE antibody which had been pre-adsorbed with rhPE (200 µg/ml) for 2 hours at room temperature. After a second blocking step with 3% BSA, PMNs were incubated with FITC-labeled goat anti-rabbit secondary antibody (1∶12,000 in PBS/1% BSA) for 1 hour. Nuclei were stained with Hoechst (1∶2000) and PMNs examined by immunofluorescence microscopy.

### PE activity assay

Freshly isolated PMNs (10^6^ cells) were stimulated with indicated reagents. Supernatant and cell lysates (lysated with 50 mM HEPES (pH 7.4), 150 mM NaCl, 15 mM MgCl_2_, 1 mM EDTA, 10% glycerol and1% Triton-X 100 in Milli Q water) were harvested and frozen until use. The protein concentration of each lysate was assayed using the Pierce BCA protein assay kit standardized to BSA according to the manufacturer's protocol (Thermo Fisher Scientific, Rockford, IL). PE activity was measured in these supernatants and lysates using the fluorogenic substrate Z-Gly-Pro-7-amido-4-methylcoumarin (2-G-P-AMC) (Bachem). Twenty microliters of cell lysate or supernatant was added to each well in a black 96-well flat-bottom plate, followed by addition of 80 µl of assay buffer (25 mM Tris, 0.25 M NaCl, pH 7.5, 2 mM DTT) containing 100 µM substrate Z-Gly-Pro-AMC. The fluorescence from liberated AMC was monitored every 1 min over 60 min at 37°C using a Fluostar reader at excitation wavelength of 355 nm and an emission wavelength of 460 nm. Fluorometric intensities observed were converted to pmol AMC released per minute using appropriate AMC standard curves.

### Western blotting

Freshly isolated PMNs (10^6^ cells) were stimulated for 9 hours with indicated reagents. Supernatant and cell lysates (lysated with 50 mM HEPES (pH 7.4), 150 mM NaCl, 15 mM MgCl_2_, 1 mM EDTA, 10% glycerol and1% Triton-X in Milli Q water) were harvested and frozen until use. Equal amounts of proteins of boiled nonreduced samples were separated electrophoretically (SDS-PAGE 10%) and transferred onto nitrocellulose membranes. The membranes were blocked with PBS-0.05% Tween-20 (PBST) containing 5% milk proteins for 1 hour at room temperature. After blocking, primary antibody rabbit anti-human PE (1∶500) in PBST containing 5% milk proteins was applied overnight at 4°C. Subsequently, the membranes were incubated with goat anti-rabbit-HRP antibodies (1∶2000) in PBST containing 5% milk proteins for 1 hour. The antibodies were visualized using commercial ECL reagents and exposed to photographic film.

### Immunohistochemistry

Paraffin sections of human lung specimens were deparaffinized, endogenous peroxidase activity was blocked with 0.3% H_2_O_2_ in methanol for 30 minutes at room temperature and rehydrated in a graded ethanol series to PBS. For antigen retrieval, the slides were boiled in 10 mM citrate buffer (pH 6.0) for 10 minutes in a microwave. The slides were cooled down to room temperature, rinsed with PBS (3×) and blocked with 5% goat serum in 1% bovine serum albumin in PBS for 30 minutes at room temperature. Sections were incubated with the primary antibody (rabbit-anti-PE, 0.6 ug/ml) in 1% bovine serum albumin/PBS overnight at 4°C. The slides were rinsed with PBS (3×) and incubated with the biotinylated secondary antibody (1∶200) in 1% bovine serum albumin/PBS for 45 minutes at room temperature. The slides were rinsed with PBS (3×) and the biotinylated proteins were visualized by incubation with streptavidin–biotin complex/horseradish peroxidase for 45 minutes at room temperature, followed by 0.015% H_2_O_2_/0.05% diaminobenzidene/0.05 M Tris–HCl (pH 7.6) for 10 minutes at room temperature. Sections were counterstained with Mayers' haematoxylin, dehydrated and mounted in Permount. Negative controls without the primary antibody and normal rabbit IgG were included as controls. Photomicrographs were taken with an Olympus BX50 microscope equipped with a Leica DFC 320 digital camera.

### PGP generation assay

Freshly isolated PMNs (10^6^ cells) were incubated with 15 µl of a 1 mg/ml solution of type I or type II collagen in PBS containing bestatin (50 µg/ml) and indicated reagents for 16 hours at 37°C. The collagen was extensively dialyzed beforehand to remove PGP. After incubation, samples were 10 kDa filtered, washed with 40 µl of 1 N HCl, and analyzed by ESI-LC-MS/MS for levels of N-ac-PGP.

### Electrospray ionization liquid chromatography–MS/MS (ESI-LC/MS/MS) for PGP and N-ac-PGP detection

PGP and N-ac-PGP were measured as previous described [34] using a MDS Sciex (Applied Biosystems, Foster City, CA) API-4000 spectrometer equipped with a Shimadzu HPLC (Columbia, MD). HPLC was done using a 2.0×150-mm Jupiter 4u Proteo column (Phenomenex, Torrance, CA) with *buffer A* (0.1% HCOOH) and *buffer B* (MeCN+0.1% HCOOH): 0 min–0.5 min 5% *buffer B*/95% *buffer A*, then increased over 0.5–2.5 min to 100% *buffer B*/0% *buffer A*. Background was removed by flushing with 100% isopropanol/0.1% formic acid. Positive electrospray mass transitions were at 270–70, 270–116, and 270–173 for PGP and 312–140 and 312–112 of N-ac-PGP.

### Statistical analyses

For all statistical analyses, GraphPad Prism version 4.0 was used. When data passed the normality test; two-tailed Student *t*-tests were used for comparing control and CSE paired groups and one-tailed Student *t*-tests were used for comparing control and N-ac-PGP paired groups. For comparing three or more paired groups, parametric data were analyzed using a repeated measures ANOVA followed by Tukey post hoc analysis. When data did not pass the normality test; Mann-Whitney tests were used for comparing two groups and Friedman tests followed by Dunns post hoc analysis were used for comparing three or more groups. Data were considered significant at p<0.05. All results are expressed as means ± S.E.M.

## Supporting Information

Figure S1 **MMP9 release by healthy donors and COPD patients.** Freshly isolated PMNs (10^6^ cells) were stimulated for 9 hours with indicated reagents after which MMP9 levels were determined in the supernatants. CSE induced the release of MMP9 from neutrophils of healthy donors and COPD patients. Neutrophils from healthy donors (n = 6) do not release significantly higher levels of MMP9 when compared to the neutrophils from the COPD group (n = 7).
(TIF)
