# Correction: Cigarette Smoke-Induced Collagen Destruction; Key to Chronic Neutrophilic Airway Inflammation?

**DOI:** 10.1371/annotation/0faf4c12-4fd4-474c-b5a4-16e49370aff3

**Published:** 2013-10-01

**Authors:** Saskia A. Overbeek, Saskia Braber, Pim J. Koelink, Paul A. J. Henricks, Esmaeil Mortaz, Adele T. LoTam Loi, Patricia L. Jackson, Johan Garssen, Gerry T. M. Wagenaar, Wim Timens, Leo Koenderman, J. Edwin Blalock, Aletta D. Kraneveld, Gert Folkerts

The affiliations for Dr.Leo Koenderman and Dr. J. Edwin Blalock are incorrect.

Dr. Koenderman is associated with: Department of Respiratory Medicine, University Medical Center Utrecht, Utrecht, The Netherlands.

Dr. Blalock is associated with: Division of Pulmonary, Allergy and Critical Care Medicine and UAB Lung Health Center, Department of Medicine, University of Alabama at Birmingham, Birmingham, Alabama, USA. 

