# Supplementary figures and images for: Cigarette Smoke-Induced Collagen Destruction; Key to Chronic Neutrophilic Airway Inflammation?
Source: PLoS One. 2013 Jan 31;8(1):e55612. doi: 10.1371/journal.pone.0055612 (PMC3561332; doi:10.1371/journal.pone.0055612)

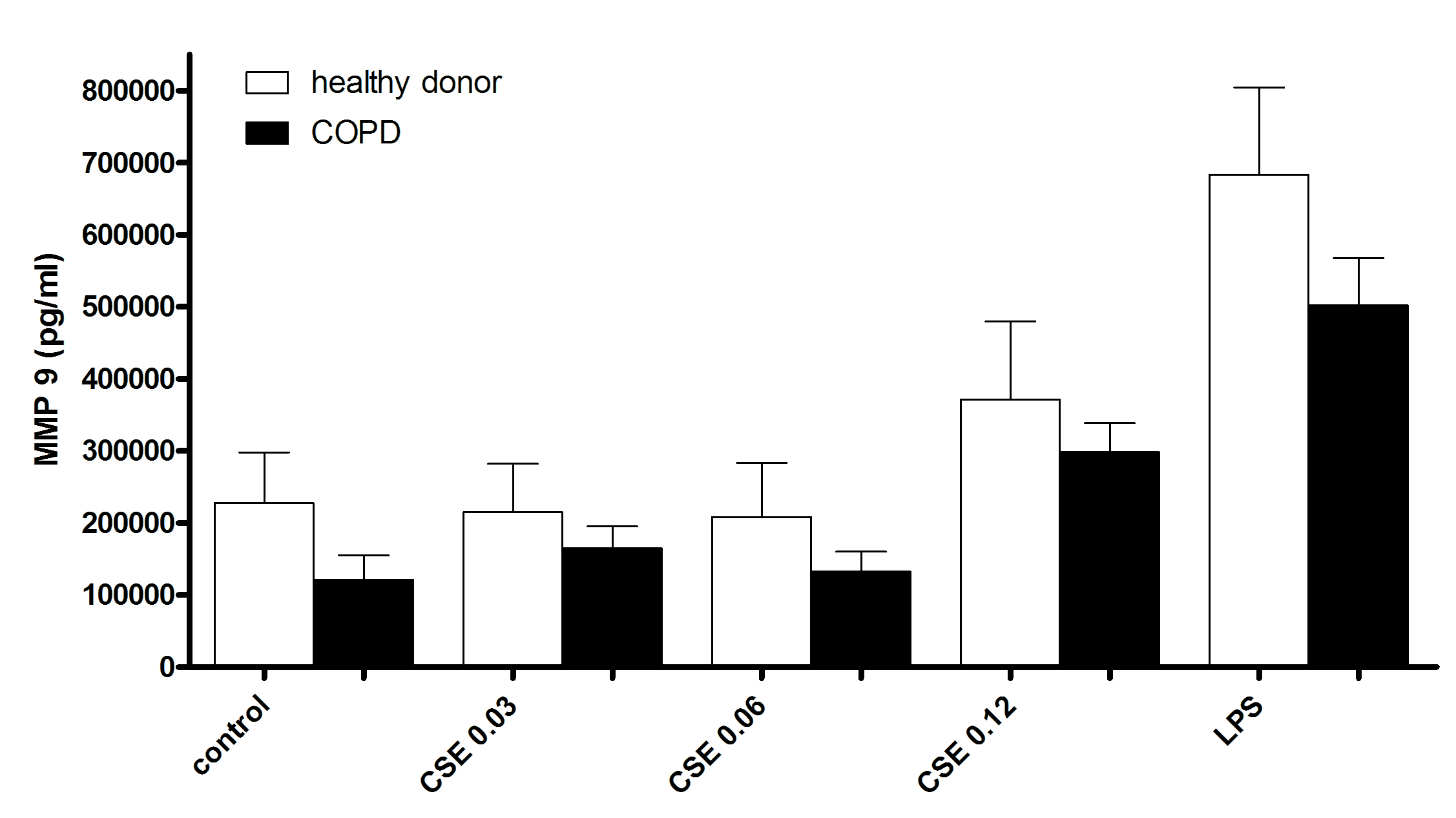

Supplement: Figure S1 — MMP9 release by healthy donors and COPD patients. Freshly isolated PMNs (106 cells) were stimulated for 9 hours with indicated reagents after which MMP9 levels were determined in the supernatants. CSE induced the release of MMP9 from neutrophils of healthy donors and COPD patients. Neutrophils from healthy donors (n = 6) do not release significantly higher levels of MMP9 when compared to the neutrophils from the COPD group (n = 7). (TIF) [file pone.0055612.s001.tif]
